# Looking at nerves, seeing the mind: Yu-Chuan Tsang as a modern Chinese physiological psychologist

**DOI:** 10.1007/s13238-020-00798-4

**Published:** 2020-11-10

**Authors:** Shiying Li, Wei Chen, Shengjun Wen

**Affiliations:** 1 Center for Brain, Mind, and Education, Shaoxing University, Shaoxing, 312000 China; 2 Department of Cognitive Neurology, Hertie Institute for Clinical Brain Research, University of Tübingen, 72076 Tübingen, Germany

**Figure 1:**
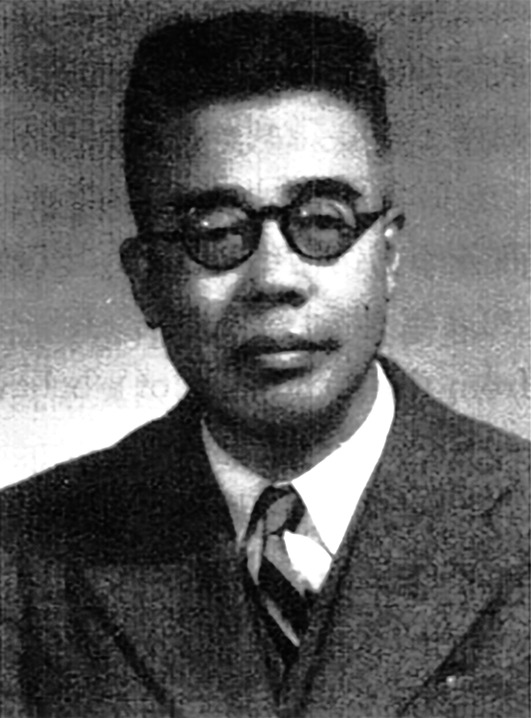
Tsang Yu-Chuan in 1954

Yu-Chuan Tsang (臧玉洤, 1901–1964), styled Botan (伯潭), was an outstanding Chinese physiological psychologist, a comparative neuroscientist, and a neuroanatomist (Fig. 1). He was a member of the First Council of the Chinese Society for Anatomical Sciences, an executive member of the second, third, fourth and fifth councils, and the Chairman of the Third Council. He was one of the founders of neuroanatomy in modern China and the Beijing Medical College (now the Department of Medicine at Peking University). Neuroanatomy is a discipline that consists of making observations and making recordings. Moreover, there are different interdisciplinary subjects based on neuroscience, including cognitive science, neurobehavioral science, neuroengineering, and psychology. Tsang’s academic career followed an opposite direction, starting from psychology and turning to neuroanatomy, where he made remarkable achievements.

**Figure 2:**
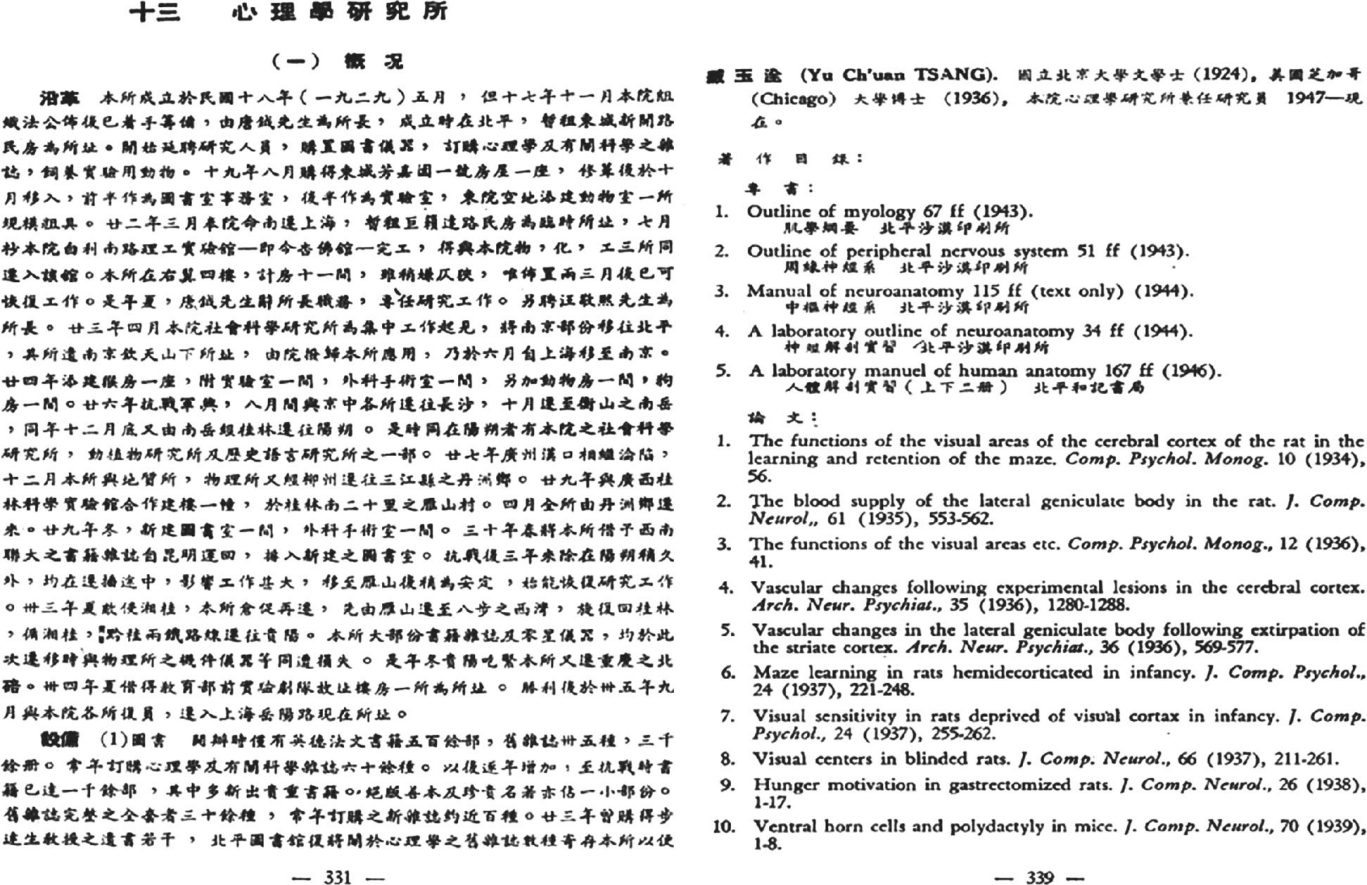
A brief introduction to Tsang as a part-time researcher at the National Research Institute of Psychology, Academia Sinica (Liu and Sun, 2008)

Tsang was born in Dongyang Ge village, in Wan County of Hebei Province on May 10th, 1901. He became a student of the Chinese Department at Beijing University in 1918, where he later changed to the Philosophy Department. After graduating from Beijing University in 1924, he worked as a compiler at the Shanghai Commercial Press. In 1926, he joined the Psychology Department of Tsinghua University as a teaching assistant. In 1929, Tsang was offered an opportunity to study in the United States, paid for by Hebei Province. In the United States, he studied in the Department of Neuroanatomy at the University of Chicago under Professor Charles Judson Herrick, the founder of comparative neurology (O’Leary and Bishop, 1960) and comparative neurophysiology (Bullock, 1983). In 1934, he completed a study entitled “Functions of visual areas in the rat cerebellar cortex in maze learning and retention”, and obtained a PhD. During his stay in the United States, Tsang devoted himself to basic theoretical research in comparative psychology and neuroanatomy and published several papers in top international journals. In 1936, Tsang returned to China after visiting and studying in England, France, Germany, and Italy, to serve as Professor in the Department of Psychology of Tsinghua University. In 1937, after the “Lugou Bridge Incident”, he joined the Department of Anatomy at the Beijing Union Medical College, where he worked as a lecturer in human anatomy and neuroanatomy. Then, Tsang gradually turned the focus of his research to neuroanatomy. He translated many classic monographs, including “the Neurological basis of animal behavior and the smell, taste, and allied sense in vertebrates”. At the end of the Anti-Japanese War in 1945, Tsang declined an invitation by Chen Hsueh Ping (陈雪屏) to devote himself to writing books. Instead, he served as Professor at Beijing Medical College until 1949. He also was a part-time researcher at the National Research Institute of Psychology of Academia Sinica from 1947 to 1949 (Fig. 2). In 1950, a preparatory office was set up by the Institute of Psychology of the Chinese Academy of Sciences. Tsang, together with other notable psychologists in China, such as Shuh Pan (潘菽), Yueh Tang (唐钺), Kuo-Hua Sun (孙国华), and Siegen Keng Chou (周先庚), were selected as members of the preparatory office. On April 27th, 1964, Tsang died of a sudden coronary artery embolism at the age of 64.

Tsang became interested in the new trend of behaviorism in early American psychology when he was still a student of the Philosophy Department at Beijing University. In 1924, his article, “The behavioral psychology of philosophy”, introduced the core aspects of behavioral psychology to China. Tsang endorsed behaviorism and believed that scholars who wavered between behavior and consciousness were making the error of “dualism”. He pointed out that behavior is an attentional process; “complex behaviors are naturally organized from simple reactions that are often not immediately connected to a higher-level combination. They combine slowly, which is the process of consciousness” (Tsang, 1924, p. 11).

**Figure 3:**
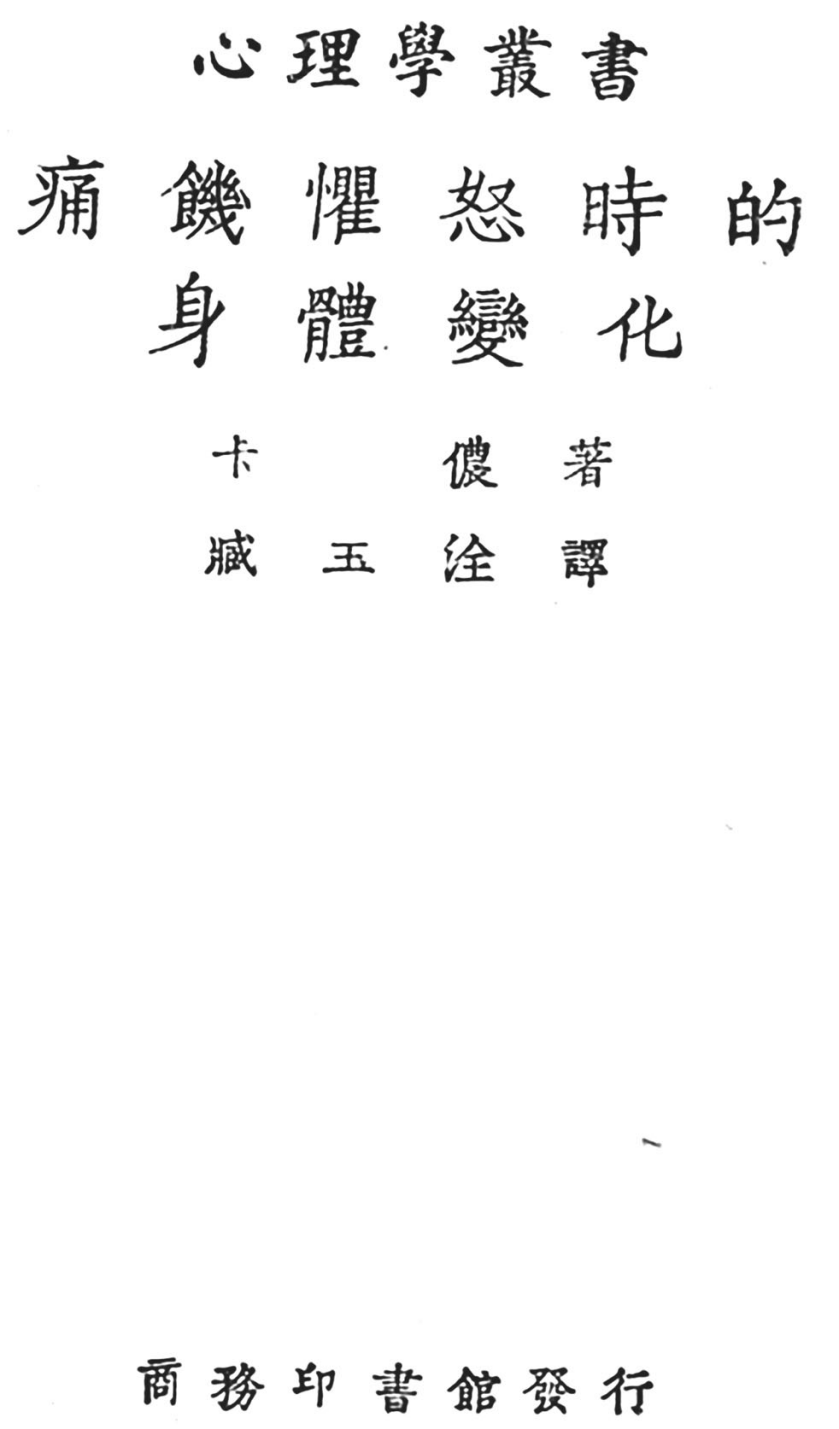
The cover of *Bodily Changes in Pain*, *Hunger*, *Fear*, and *Rage*

In 1925, Tsang translated and published “Psychology from the standpoint of a behaviorist”, written by John Watson, an American psychologist who founded behavioral psychology. The book included Watson’s “Behaviorism: A new annotation of psychology” and McDougall’s “Principles of Psychology-Behavioral criticism”. Zing-Yang Kuo (郭任远) wrote the foreword and McDougall wrote the postscript (华生, 1925). The introduction and reflection on behavioral psychology led Tsang to investigate the biological basis of psychological phenomena, which established his academic relevance for 30 years. His academic relationship with Professor Walter Bradford Cannon, an internationally renowned physiological psychologist, is an important story for the history of neuroscience. Cannon’s famous work, “Bodily changes in pain, hunger, fear, and rage”, published in 1915, established his place in the history of physiological psychology (Fig. 3). The main idea of this book is that “fear, anger, pain, and hunger are all primitive experiences shared by human beings and lower animals, and these experiences dominate human and animal movements. Therefore, it is of general and fundamental importance to understand the physiological conditions accompanying these experiences for explaining behaviors. Physiological adaptation and interpretations are interesting, not only for physiologists and psychologists but also for others. Therefore, it is worth collecting the reports of experiments that were originally published in the American Journal of Physiology and Medicine (Cannon, 1928, p. 234). In 1925, Tsang began translating Cannon’s book into Chinese, which was published by the Shanghai Commercial Press in 1928 (Cannon, 1915; 1928). As the translator, Tsang sent two copies of the Chinese version of the book to Cannon, accompanied by a letter that described his experience of farming for generations, bereavement of his father as a teenager, and finishing his studies in Beijing University with a strong desire for knowledge and frugality. He has already translated several important English monographs on psychology, and at the time, he was translating a psychological work in French. He yearned to go to the United States because of their advanced instruments that allow precise experiments, which were not available in China. He also included a photo with his letter. Cannon not only wrote back to encourage Tsang to conduct his academic research, but also sent a revised version of “Bodily changes”. Correspondence between Tsang and Cannon continued until the outbreak of the Anti-Japanese War. Whenever Tsang changed his place of study or started a new job, he always wrote to Cannon, who was also informed about his research. There are over 20 originals and copies of their correspondence in Cannon’s archives. Tsang minimized the use of professional terms or additional explanations when translating to accommodate readers who were less familiar with medicine.

During his studies in the United States under the careful guidance of Herrick, Tsang conducted a series of comparative psychological studies on animal vision, hunger motivation, as well as neuropsychological studies and achieved remarkable results. This led him to gain an international influence. At that time, the concepts of Karl Lashley, a famous American physiological psychologist, occupied the leading position in the study of neurophysiology of behavior. Lashley proposed that the brain works as a whole and that there are no discrete brain regions specific to certain functions: a function is not located in the brain but is a characteristic of the whole brain. Mental functions require a certain amount of brain but it does not matter which part of the brain is intact (Lashley, 1929). Lashley summarized his findings with two principles: (1) overall activity, which suggests that the cerebral cortex works as a unified whole because the loss of capacity after partial destruction of the cortex is more closely related to the amount of damage than to the location of the damage; (2) equipotentiality, which suggests that any part of a functional brain area can perform the functions related to that functional area. For example, any cell in the visual area of the cortex can produce vision. Therefore, in order to destroy a certain brain function, it would be necessary to destroy the whole brain area related to that function. Moreover, if any part of a functional brain area remains undamaged, then the function will be maintained (Weidman, 1999).

While studying for his doctorate in anatomy at the University of Chicago, Tsang investigated the role of the visual area of the cerebral cortex in the learning and memory of rats. As expected, Tsang learned through experiments in 1934 that maze learning ability could be acquired following the ablation of the visual area of the cerebral cortex in rats. Tsang then began to study the morphological changes of blood vessels in the lateral geniculate body after removing the visual cortex. To further examine possible causes of behavioral disorders after cortical injury, he examined direct damage to neural elements, the dysfunction of cells in other regions caused by divisions, and the metabolic changes caused by vascular changes. He made specific discoveries during this research. In 1934, he published an article, “the blood supply of the lateral geniculate body in the rat”, which examined the arterial origin of the lateral geniculate body of the primary visual center and showed that the main functional areas had dense blood vessels. The study also demonstrated that the vascular density and the permeability of the lateral geniculate body increase with the enlargement and number of afferent vessels and the thickening of the capillary network. However, there were no significant vascular changes in the ipsilateral or contralateral geniculate body after eyeball enucleation of one eye (Tsang, 1934a). Today, Tsang’s research on the posterolateral geniculate body after removal of the visual cortex played an important role in forming the cornerstone for promoting the development of visual physiology research in China. Tsang’s research achievements in this field were also included in his article, “Physiology of vision in China: past and present”, by Hsiang-Tung from the Chinese Academy of Sciences (Chang, 1988).

Later, despite some turbulence and shift in his research interests, Tsang engaged in the study of the visual cortex and published an experimental report, “Visual centers in blinded rats”, in the *Journal of Comparative Neurology*. He reported that “the visual centers of rats show equal degradation at all levels when rats lose one or two eyes at different ages”. Of these, the degeneration of the superior colliculus is the most serious, followed by the lateral geniculate body, and the visual cortex. The degree of degeneration depends on the intimacy with omentum, the duration of loss of vision, and the loss of one or two eyes” (Tsang, 1936b, p. 246). In 1950, Tsang further compared the visual centers of rats and moles and found that moles living underground have extremely small eyeballs, where the visual centers have degenerated at all levels and the degree of degeneration depended on the relationship between the center and the retina. This was an example of lifestyle affecting organ shape (Tsang, 1950). Unfortunately, Tsang only found limited changes in the visual system at the level of anatomical structures, cells, and the environment and did not systematically research and summarize the visual system at the theoretical level. Over 30 years later, David H. Hubel and Torsten Nils Wiesel conducted similar research and conducted a series of critical experiments in the field of visual development. In their visual deprivation experiments, they sewed shut the eyes of newborn cats and discovered that the dominant functional column in the cortex has a crucial influence in processing sensory information. Eventually, based on a series of studies, they concluded that visual processing in the cerebral cortex of animals is hierarchical. These important discoveries in physiology led to them being awarded the 1981 Nobel Prize for medicine and physiology.

In 1937, Tsang reported his experimental finding that brain function related to maze learning in rats is non-localized, in a paper entitled, “Maze learning in rats that were hemi-decorticated in infancy”. In this experiment, Tsang conducted four types of hemidecortications on the cerebral cortex of infant rats and discovered that regardless of type, hemi-decortication always resulted in the deterioration of field of vision. This shows that each part of the cerebral cortex has certain generalized non-local functions, in addition to its unique local functions. When part of the cerebral cortex is damaged, the generalized facilitative forces of the remaining parts quickly reorganize into a dynamic model to meet the demands of the environment. Tsang also noted in his experiments that the efficiency of brain tissue depends on the integrity of various parts of the cerebral cortex. The efficiency of two widely isolated parts is less than that of the single parts of the brain with the same quality; isolated parts lack mutual promotion (Tsang, 1937a). These conclusions of the experiments conducted by Tsang are consistent with the principle of overall activity, proposed by Lashley. In addition, Tsang’s experiments reported in this paper support the conjecture that damage to the cerebral cortex in childhood is far less serious than in adulthood. Tsang selected 79 newborn rats, according to strict experimental requirements, and trained them to walk a maze by using hunger as the driving force for learning. Six different combinations of hemidecortications were conducted, removing half of the cortex of the newborn rats in the experimental group, and their learning ability was tested after growth of age. The results indicated that even extensive cortical damage in infant rats had relatively little impact on maze learning, which confirmed his previous hypothesis. The results of this experiment were consistent with the notion that a rat’s maze performance is a common function that is not affected by specific parts of the brain, as suggested by Lashley in “Brain mechanisms and intelligence: a quantitative study of injuries to the brain.” (Lashley, 1929). His article was published in the *Journal of Comparative Psychology*. Tsang’s conjectures and conclusions in this empirical study have contributed to contemporary clinical medicine. Tsang’s studies suggested that infant’s brains have compensatory plasticity and thus brain areas that are the focus of epilepsy can be removed from the brains of children. Today, children before the age of 10 years can have hemispherectomies as a treatment for epilepsy and live normally into adulthood.

Tsang’s experiments indicated that very few rats had partial pattern vision after dissecting the striatum area of the rat brain, which is very important region for pattern vision. Tsang suggested that this might be caused by the gradual differentiation of pattern and brightness vision. Pattern vision is lost when the rats’ visual cortex is damaged in infancy, and brightness vision might develop excessively due to compensation to regulate basic types of patterns (Tsang, 1937b). This experimental phenomenon might also partially demonstrate the principle of equipotentiality described by Lashley. Tsang’s work on animal vision has aroused the interest of many neuroscientists. Lubar, Schostal, and Perachio conducted experiments on cats, which suggested that the striatal cortex of cats is involved in the analysis of spatial orientation (Lubar, 1967). They found that a small injury limited to the striatal cortex did not interfere with visual pattern perception but led to a serious deficiency in the ability to develop an avoidance response toward a buzzer that was used as a conditioned stimulus in a shuttle box.

Tsang also conducted research on the visual cortex of rats. At the time, most reports on functional preservation or recovery after early damage to the cerebral cortex were based on damage to the neocortex. Lashley (1929, 1943) and Tsang (1934b, 1936a) found that damage to the neocortex (centered on the 17 areas, human visual cortex includes striate cortex and extrastriate cortex, and 17 areas represents striate cortex) of rats caused more deficits than damage to the optic radiation or peripheral blinding by enucleation in acquiring and maintaining behavioral ability in the complex Lashley III maze. Moreover, Lashley showed that combining enucleation and lesioning of the posterior neocortex resulted in even greater deficits (Lashley 1943). Tsang (1936a) reported that cortical lesions could also produce deficits in previously enucleated rats. These results showed that the visual area of the posterior cortex participates in the learning of complex mazes in rats with intact vision; furthermore, the visual area of the posterior cortex also affects the spatial orientation of rats with visual unavailability. The conclusion of this experiment is consistent with the principle of equipotentiality, described by Lashley (Hein and Jeannerod, 1983). Later et al. (1960) also conducted experimental studies on the cerebral cortex: their experimental results showed that the delayed effect of injury to the same area in different parts of the cerebral cortex was identical for different types of learning. They interpreted the results as suggesting that all regions of the cerebral cortex have a general promoting effect on other areas, in addition to their specific functions. This conclusion was tantamount to Lashley’s principle of equipotentiality.

In 1938, Tsang continued to conduct experiments on rats and further published a paper entitled, “Hunger motivation in gastrectomized rats”, in the *Journal of Comparative Psychology*. In this study of rats with gastrectomy, he developed two independent variables consisting of injections of environmental and nutrient solutions. He used body weight and task performance of newborn rats as the dependent variables and used a control group to strictly control for the experimental variables. The results showed that gastrectomized rats performed similarly to normal rats in the initial task following a day of starvation and that the mice were just as active even without a contractile stomach. The gastrectomized rats became significantly less active than normal rats only during the later stage of the task. Gastrectomy may have an inhibitory effect on general activity because the rats could not actively complete the experimental task, though hunger may have been present because it is unrelated to the presence of a digestive system. Moreover, gastrectomized rats were three times more active one hour before feeding than after feeding, which was indicative of the driving force of hunger and the calming effect of food on the intestines (Tsang, 1938). This experiment also demonstrated to some extent that certain types of hunger might exist in people who have had stomach ulcers or cancers removed (Myers, 2010). Tsang also studied hunger in gastrectomized rats by using different experimental research methods. Ultimately, Tsang concluded that the motivation of gastrectomized rats measured in maze running and activity cages was not different from that of rats with a normal stomach. Therefore, it cannot be concluded that hunger motivation is entirely caused by stomach contractions (Pronko and Bowles, 1952). Zhentong Mei (梅镇彤), a Chinese physiologist, commented: “The experiments of Yu-Chuan Tsang are the first to use experiments to study animal behavior in China, and at the same time, it has also created a precedent in our country to study behavioral changes as an index in the study of cerebral cortex functions.” (梅镇彤, 2013). We regard these works as early representations of Chinese achievements in the field of comparative psychology, which—together with the research of Lashley and Cannon—promoted the wave of physiological psychology in the early 20th century.

Tsang was also one of the early modern Chinese psychology scholars who focused on the classical conditioning reflex theory of the Soviet physiologist I. Pavlov and introduced it to the academic circle in China. As early as 1929, he discussed Pavlov’s conditioned response alongside his research work and summarized the significance of Pavlov’s work. First of all, we need to get rid of psychological conjectures before study the physiological reflexes of the brain. Pavlov turned the study of psychology into the exclusive product of psychologists. Secondly, Pavlov used the saliva secretion reflex as a research medium, a skillful technique, which became the model for animal research methods. Finally, Pavlov used dogs to study psychosis, suggesting that the disorder of brain function is the cause of the disease. Of course, as an independent scientist, Tsang put forward a critique of Pavlov’s research. Firstly, Tsang (1929) considered that “The materials of Pavlov’s book were all products of Pavlov’s laboratory, although researchers other than Pavlov and his disciples have also investigated the same reflex.” Secondly, studying the functions of the brain through the glandular salivary secretion reflex was unconvincing. Thirdly, the research of Pavlov and his disciples has no priority, and the results from decades of different situations were mixed together, which made distinguishing specific information difficult (Tsang, 1929).

**Figure 4:**
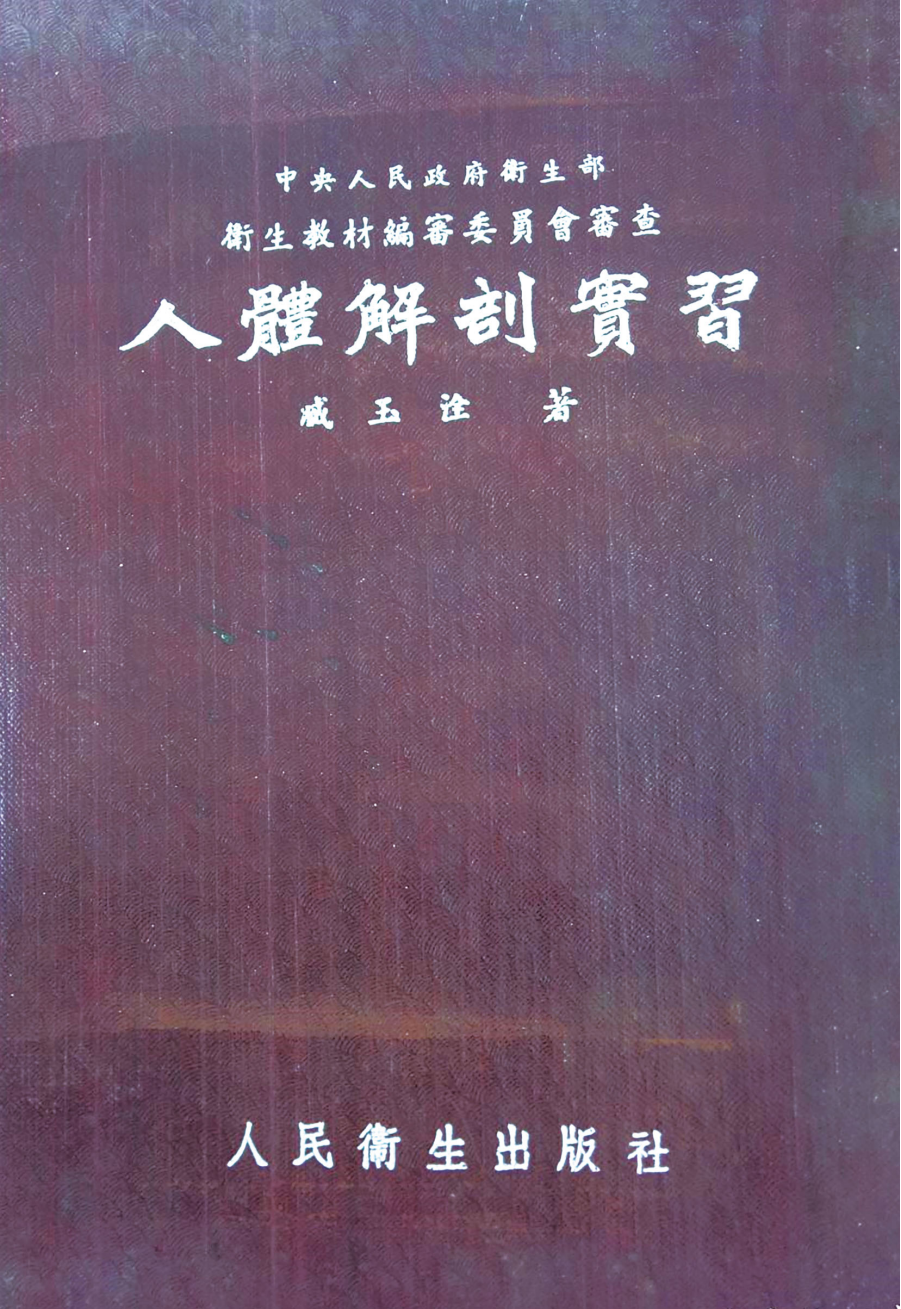
The cover of *Human Anatomy Practice*

In addition to his remarkable academic achievements, Tsang’s professionalism is particularly commendable. In April 1942, Tsang suffered from typhus fever. When his body had recovered a little, he immediately devoted himself to his work. He compiled and published several teaching handouts, which were later reprinted many times, including the “Central Nervous System”, “Neuroanatomy Practice”, and “Human Anatomy Practice”, among others (Fig. 4). In 1950, he offered several advanced classes for senior teachers, trained many neuroanatomy professionals, and accompanied the motherland’s neuroanatomy cause through difficult years. In 1951, Tsang fell more ill than before, though he continued to write while struggling with his health. In 1955, he enrolled the first cohort of graduate students who majored in neuroanatomy, following the founding of the People’s Republic of China. Tsang was familiar with English, French, German, Spanish, Russian, and Esperanto. He stated that experience is persistence. Although he only learned the German alphabet and Pinyin before studying abroad, his habit of reading for a few minutes every day allowed him to master the language. He also mastered Russian after turning 50 years old. Because of his profound professional background and excellent foreign language skills, Tsang was able to lead a group of young teachers in translating specific chapters of “Human Neuroanatomy”, “Human Anatomy Atlas”, and “Neurological Foundations of Animal Behavior”, written by his mentor Herrick (which was entitled “Evolution of Nervous System” and some of his other famous neuroscience studies).

The motto, “The day is short, but the work is much”, facilitated Tsang’s outstanding academic achievements. In 1947, the Chinese national government decided to formally establish the academician system and the Central Research Institute elected its first academician. On July 17 of the same year, Hu Shih (胡适) sent a signed letter to 119 academicians to elect the Preparatory Committee and submitted a “List of academicians proposed by Beijing University to the Central Research Institute”. Among them were three psychologists in the biological group, Tsang, Chih-Wei Luh (陆志韦), and Ging-Hsi Wang (汪敬熙). From 1949 to 1950, a comprehensive report of the national scientific expert’s investigation, initiated by the Chinese Academy of Sciences, showed that Tsang was nominated as the 15th person in the psychology group. In September 1956, Tsang was selected as one of the first grade professors by the Ministry of Higher Education, which confirmed that his peers unanimously recognized that his work played a central role in the century-long development of Chinese physiological psychology.
